# Supplementary material for: Modeling glioblastoma heterogeneity as a dynamic network of cell states
Source: Mol Syst Biol. 2021 Sep 16;17(9):e10105. doi: 10.15252/msb.202010105 (PMC8444284; doi:10.15252/msb.202010105)
Supplement: Supplementary file 6 — Source Data for Figure 5 [file MSB-17-e10105-s004.zip › Figure5A_sourcedata/GSEA_3017/nosigdb_stateE.GseaPreranked.1622795031093/OLSEN_MESENCHYMAL.html]

Details for gene set OLSEN\_MESENCHYMAL[GSEA]

|  || Dataset | state63017 |
| Phenotype | NoPhenotypeAvailable |
| Upregulated in class | na\_pos |
| GeneSet | OLSEN\_MESENCHYMAL |
| Enrichment Score (ES) | 0.78613704 |
| Normalized Enrichment Score (NES) | 3.7652187 |
| Nominal p-value | 0.0 |
| FDR q-value | 0.0 |
| FWER p-Value | 0.0 |
Table: GSEA Results Summary

  

Fig 1: Enrichment plot: OLSEN\_MESENCHYMAL      
 Profile of the Running ES Score & Positions of GeneSet Members on the Rank Ordered List

  

| PROBE | GENE SYMBOL | GENE\_TITLE | RANK IN GENE LIST | RANK METRIC SCORE | RUNNING ES | CORE ENRICHMENT || 1 | IGFBP5 |  |  | 4 | 1.015 | 0.0847 | Yes |
| 2 | CCL2 |  |  | 5 | 0.975 | 0.1701 | Yes |
| 3 | ID3 |  |  | 6 | 0.972 | 0.2553 | Yes |
| 4 | ANXA1 |  |  | 12 | 0.826 | 0.3224 | Yes |
| 5 | PRSS23 |  |  | 21 | 0.722 | 0.3770 | Yes |
| 6 | IFITM3 |  |  | 26 | 0.682 | 0.4326 | Yes |
| 7 | EMP1 |  |  | 40 | 0.584 | 0.4697 | Yes |
| 8 | CALD1 |  |  | 41 | 0.580 | 0.5206 | Yes |
| 9 | IFITM2 |  |  | 46 | 0.536 | 0.5633 | Yes |
| 10 | LHFPL6 |  |  | 71 | 0.451 | 0.5769 | Yes |
| 11 | COL5A2 |  |  | 83 | 0.434 | 0.6031 | Yes |
| 12 | CST3 |  |  | 86 | 0.425 | 0.6381 | Yes |
| 13 | TMSB4X |  |  | 93 | 0.412 | 0.6678 | Yes |
| 14 | CAVIN1 |  |  | 100 | 0.399 | 0.6963 | Yes |
| 15 | TIMP3 |  |  | 106 | 0.391 | 0.7251 | Yes |
| 16 | CD99 |  |  | 107 | 0.389 | 0.7593 | Yes |
| 17 | LGALS1 |  |  | 113 | 0.368 | 0.7861 | Yes |
| 18 | S100A6 |  |  | 176 | 0.312 | 0.7466 | No |
| 19 | PRRX1 |  |  | 229 | 0.278 | 0.7148 | No |
| 20 | ZFP36L1 |  |  | 287 | -0.255 | 0.6757 | No |
| 21 | EGR1 |  |  | 656 | -0.398 | 0.3132 | No |
Table: GSEA details [plain text format]

  

Fig 2: OLSEN\_MESENCHYMAL: Random ES distribution      
 Gene set null distribution of ES for **OLSEN\_MESENCHYMAL**
